# Digitalisation in accounting: a systematic literature review of activities and implications for competences

**DOI:** 10.1186/s40461-023-00141-1

**Published:** 2023-04-04

**Authors:** Julia Pargmann, Elisabeth Riebenbauer, Doreen Flick-Holtsch, Florian Berding

**Affiliations:** 1 Institute for Vocational and Business Education, University of Hamburg, Sedanstraße 19, 20146 Hamburg, Germany; 2 Institute of Business Education, University of Graz, Graz, Austria; 3 Institute of Education, University of Zurich, Zurich, Switzerland

**Keywords:** Digitalisation, Expertise, Accounting education, Competences, Activities

## Abstract

The digitalisation of processes is a current topic in accounting. New technologies can change activities which in turn may require different skills from accounting graduates. This paper aims to shed light on the changes that digitalisation brings about in various areas of accounting by assessing the types of activities (non-routine and routine) and corresponding competences in the context of progressing stages of digitalisation. In addition, it is analysed how different technologies are used in these activities and where their execution is placed within the supply chain. The systematic literature review shows a lack of expertise in the field of digitalisation that enables graduates and employees to successfully manage respective processes in the workplace. While routine activities are continuously being automated or digitalised, non-routine activities and the corresponding skills have a similarly increasing importance for employees in accounting as the acquisition of general digital competences.

## Introduction

In the context of digitalisation, it is part of everyday life for customers to purchase products and services with the aid of an application online and in physical stores. The streams of products and finances within and between companies are also highly interconnected when they are based on digitalised processes (Appelfeller and Feldmann 2018). Digitalisation thus encompasses the entire supply chain of a product or service. Accounting is one area of the company that documents these processes with customers and other companies, enables these processes, supports them technically, and connects them with internal and external interfaces (Bleiber 2019; Klein and Küst 2020). In this context, companies nowadays face the challenge to make decisions on the introduction of new technologies and digitalised business processes into the area of accounting, among others (Appelfeller and Feldmann 2018). Digitalisation can address various stages, from substitution (e.g., self-check-out counters in retail shops) to business process innovation (e.g. automated storage and payment with RFID chips; see for these examples e.g. Litfin and Wolfram 2010). It is expected that not only costs and productivity are essential decision criteria for the introduction of technologies and digitalised business processes (Ashoka et al. 2019; Chen et al. 2012) but also the extent to which employees can master and employ technologies and possess the competence to fulfil new or evolving tasks (Aepli et al. 2017; Bonin et al. 2015; Cong et al. 2018; Egle and Keimer 2017; Seeber and Seifried 2019).

In this context, it is necessary to describe how digitalisation affects the requirement profile regarding the competences of employees in accounting. On the general level, the concept of competence is discussed in many ways and often focused on mastering domain-specific tasks and requirements (Hartig et al. 2008; Weinert 2001). The types of activities themselves range from manual routine tasks (e.g. paper-based bookkeeping) to interactive activities (e.g. solving problems with automated digital bookkeeping) (Seeber et al. 2019; Seeber and Seifried 2019). Hence, in order to learn more about relevant competences in accounting in a digitalised world, we have to analyse the different tasks and actual accounting activities that result in changing competence requirements as a basis. There are insightful studies available that analyse changes and developments of domain-specific requirements and competences as well as types of activities in the context of digitalisation (Aepli et al. 2017; Iten et al. 2016; Sachs et al. 2016; Seeber et al. 2019). However, rather little information is offered by these reports when it comes to accounting and its specific activities as well as technologies as tools for these activities. Thus, we assume that knowledge about these (new) activities in accounting and the corresponding digital technologies provide a good starting point to infer possible changes in necessary competences for acting in professional situations. Thus, our research questions are:

- RQ1:Which activities are concerned with digitalisation in which areas of accounting?
- RQ2:How do accounting activities differ in their respective stages of digitalisation?
- RQ3:Where are these activities placed within the supply chain?
- RQ4:Which technologies are used to perform these activities?
- RQ5:Which advantages and disadvantages are connected to these activities?

The results aim to provoke further curricular and didactic discussions about learning and teaching accounting in vocational education and training on the one hand and higher education on the other.

Starting from a specification of digitalisation along its stages and types of activities, we explain in the following the approach for the systematic analysis based on a review of relevant literature. To actually gain insight into concrete developments of activities in accounting, we provide a systematic overview of the types of activities and the stages of digitalisation. Moreover, we consider the specific stages of digitalisation in combination with the different types of activities, as we expect differences between certain types of activities (e.g. interactive activities) and specific stages of digitalisation (e.g. innovation). In modern accounting, technologies are the main set of tools accountants use to execute their tasks, much like the tools of a craft. When technologies change, so do activities. Therefore, technologies need to be considered in this review as well. In addition, the placement of activities within the supply chain is included, as different departments require different competences and develop different digitalisation projects since the stakeholders differ. Furthermore, the advantages and disadvantages of digitalisation are to be surveyed since they offer insights why activities are digitalized.

In the German-speaking countries, one’s ability to follow an occupation successfully is described by the concept of vocational action competence. Established competence frameworks usually differentiate between professional, social, and self-competences as major dimensions with methodological and technological competences anchored horizontally across all major dimensions. To decide which competences are needed for which occupation, activities are analysed that allow these competences to be fostered. Thus, it is relevant to analyse activities in accounting to derive necessary changes in curricula. Our research systematises digitalised activities that we employ to draw conclusions for the competence development of future accountants.

## Framework for categorising digitalisation and types of activities

When discussing the consequences of digitalisation for accounting activities, at least two aspects must be considered in depth: first, the specific stages of digitalisation and, second, the types of activities.

**Fig. 1:**
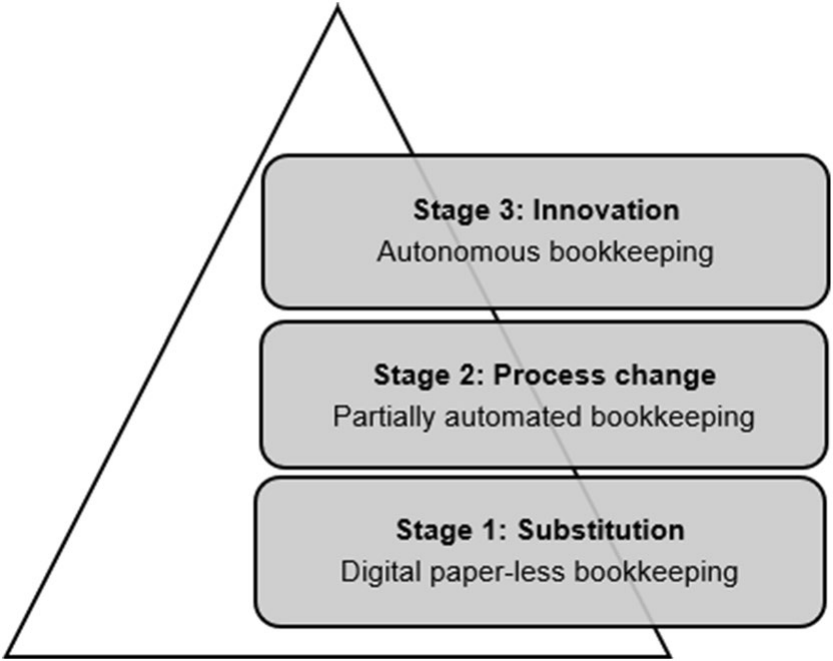
Stages of digitalisation in accounting

Figure 1 shows an overview of three different stages of digitalisation in the context of accounting, each of which is disruptive to a different degree for activities and thus competences (Bleiber 2019; Hübl 2020): *substitution*, *process change,* and *innovation*. While in stage 1 analogue data or activities are substituted by digital ones, new technologies lead to changes in processes in stage 2. Stage 3 addresses innovation, both processes and their respective outcomes are changed.

An insightful framework of digitalised activities is suggested by Aepli et al. (2017), who adopted the classification from Spitz‐Oener (2006) and validated it in expert interviews. The framework was chosen because digitalisation is more likely to change activities in a profession rather than erase it in their entirety (Aepli et al. 2017; Arntz et al. 2016; Autor 2015; Seeber and Seifried 2019; World Economic Forum 2018). In addition, some activities are more prone to digitalisation than others.

Table 1 illustrates this approach using types of activities (left column) which are intended to provide deeper insight into the facets of potentially digitalised activities with generic examples (middle column) and specific accounting-related examples (right column).

**Table 1:** Types of activities (Aepli et al. 2017, 41, examples freely translated and supplemented)

Types of activities	Generic application for different professions	Application in accounting
Manual routine activities	Sort, pack, and ship products and goods	Physically sort and scan invoices and receipts, enter standard invoices and receipts in an accounting program, print analyses
Cognitive routine activities	Data management, check invoices, process transactions	Book incoming invoices, check recorded invoices online, complete incomplete bookings
Analytical non-routine activities	Preparation of teaching and learning processes; planning activities; data preparation, interpretation, and presentation	Analyse monthly and year-end financial reports, develop a plan to improve the dunning process
Manual non-routine activities	Maintain and service machines and equipment	Set up interfaces to customer systems, solve problems with the automatic paper feed of the scanner for the invoices
Interactive non-routine activities	Provide information, teach, advise, instruct	Explain the annual statement to stakeholders, renegotiate payment conditions, internal exchange with the tax advisor

Based on Dengler et al. (2014), we suggest to include automated routine activities, even though they are not performed by professionals (e.g. the automatic import of outgoing invoices from invoicing to accounting). Due to the increasing number of automated workflows in accounting, this adaptation provides the ability to better record and illustrate these changes as they reflect all activities that take place in the different areas of accounting. Thus, the six types of activities provide a comprehensive and differentiated range of activities. This categorization of activities will serve as a framework for our literature review in order to establish comparability and transferability to other (commercial) fields and professions as well.

## Research design and methodology

**Fig. 2:**
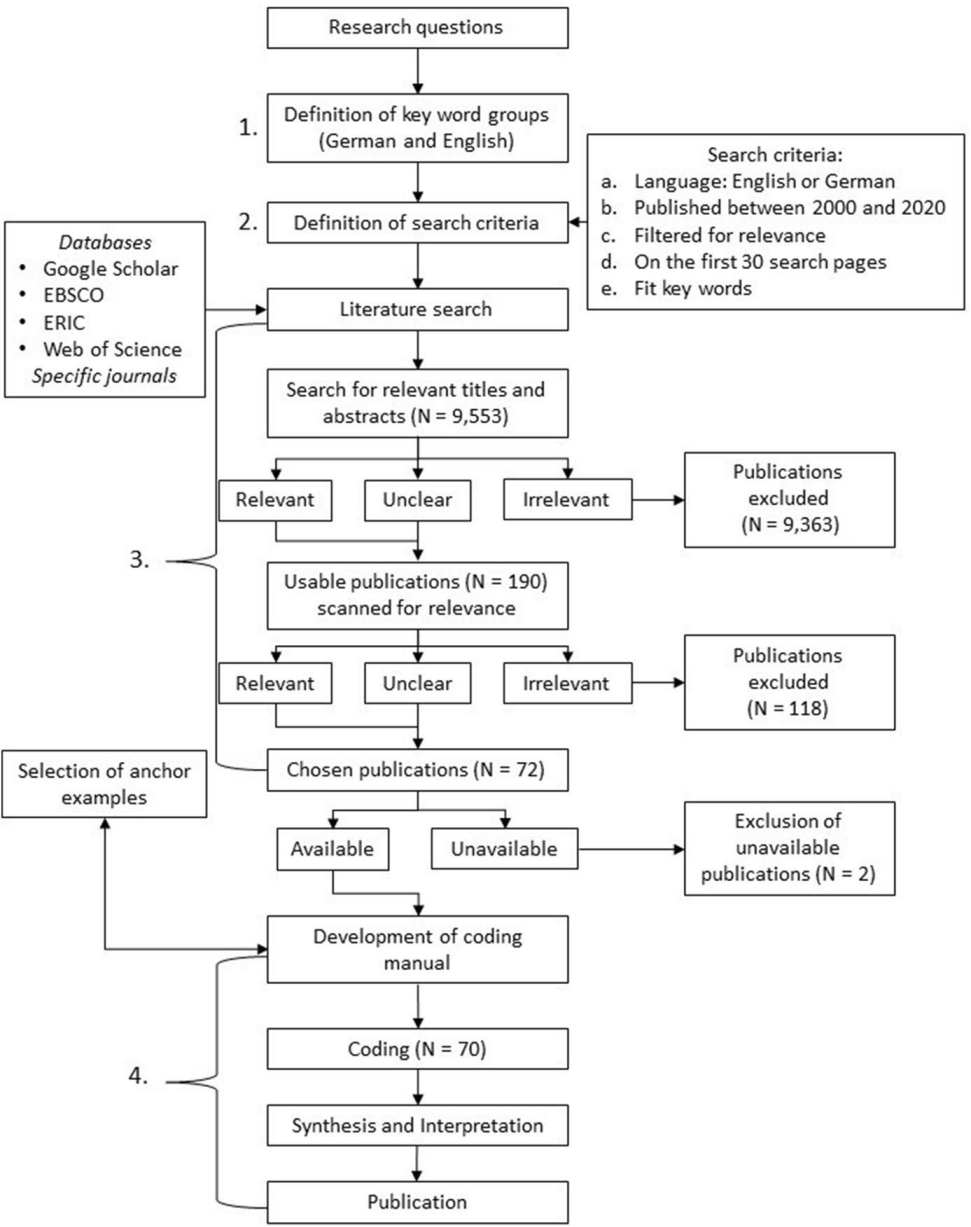
Flow chart of research design

A systematic literature review was conducted to find out how the accounting activities of future accounting professionals have changed due to digitalisation. The four-step process (Fig. 2) started with (1) the definition of up to fourteen keyword groups consisting of technical search terms in both English and German, which for example include “computer-assisted,” “data processing,” “technological speed of change,” “digitalisation rate,” “ERP,” “enterprise-resource-planning,” “accounting software,” “OCR,” “optical character recognition,” “big data,” “blockchain,” or “process mining.” Since the choice of keywords directly impacts the search hits, we used four strategies to ensure that we were able to use as many relevant terms as possible: (a) We conversed with an accounting professor about trends and current developments regarding digitalisation in the field, (b) we scoped the databases that we used for our full search and analysed the most frequently occurring trends and technologies, (c) we analysed accounting curricula from vocational education and higher education institutions to adjust our concepts of different competence frameworks, and (d) we used a synopsis from different curricula and textbooks to systematise activities. The keywords were grouped because of technical limitations regarding the valid number of connectors, particularly in Google Scholar. To ensure comparability between databases, we used these groups throughout our search.

After the definition of suitable terms, (2) a search was performed in the *Google Scholar*, *ERIC*, *EBSCO,* and *Web of Science* databases and complemented by specific relevant international journals (“Journal of Accounting Education,” “Journal of Emerging Technologies in Accounting,” “Journal of Information Systems”) which were chosen due to their topicality regarding the research questions. They were selected based on their impact and the number of search hits. Due to the increasing speed of digitalisation, the period between 2000 and 2020 was selected. In the later stages of the study, the research group also decided to include a 2021 article due to its topical suitability.

While performing the content analysis, we applied a strategy that limited the number of hits. Only the first 30 pages of search hits for each keyword group were scoped for relevance. This was necessary because of the broad scope of the search terms. This specific cut-off point was chosen because, in a preliminary scope, the number of potentially useful search hits declined drastically. On average, no more (potentially) relevant hits were identified after 29 to 30 pages, thus setting this number for the full search as well. This process yielded a total of 9,553 potentially relevant hits across all media. A scan of both titles and abstracts resulted in (3) a total of 190 potentially suitable sources of which 72 articles proved relevant to the research questions. Irrelevant publications either referred only to accounting *or* digitalisation, were purely didactic, or had a general IT-orientation without an accounting focus. If the relevance could not be decided, the full text was scanned for the connection between accounting and digitalisation. The last step (4) was the content analysis. A total of 70 articles were retrievable and thus used in the coding process. The coding manual was developed and completed with appropriate anchor examples to illustrate the variety of possible accounting activities and to align our general understanding of the categories. The coding ensued in pairs to promote discussion.

### Analytical framework

**Fig. 3:**
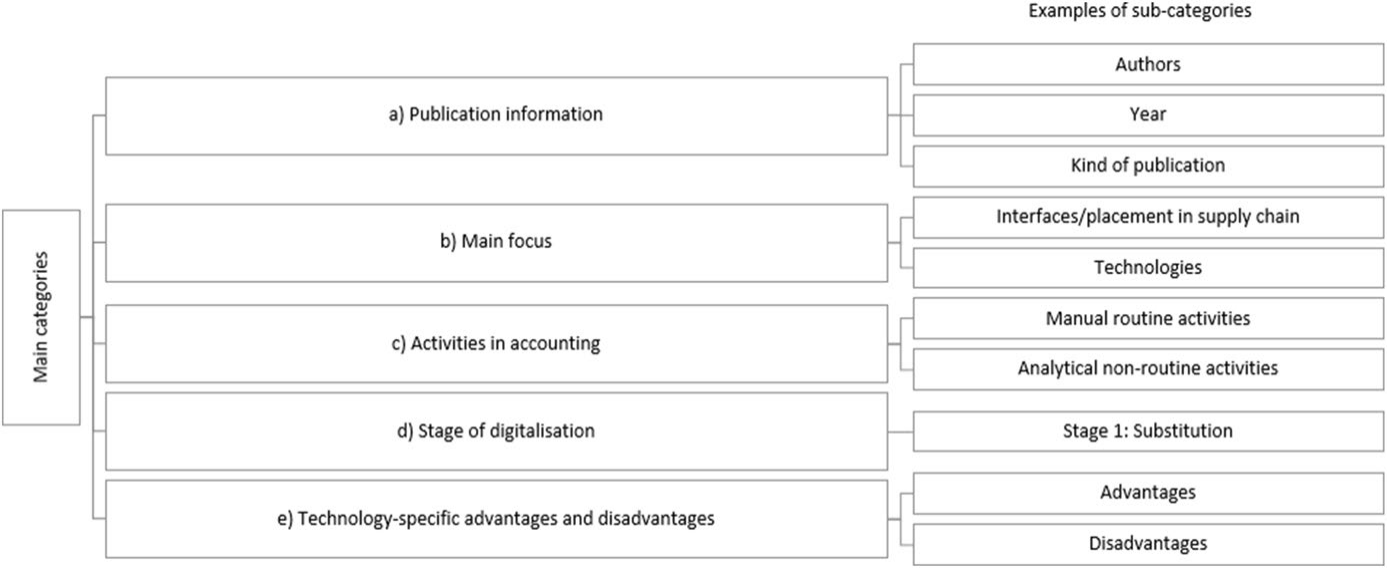
Coding tree with sample sub-categories

The coding tree (Fig. 3) consists of (a) publication information, (b) the publication’s main foci and (c) types of activities (automated, manual and cognitive routine activities, manual and analytical and interactive non-routine activities) in different areas of accounting (financial accounting, controlling, balancing, financial forecasting, managerial accounting, ‘other’). Moreover, the coding tree included (d) respective digitalisation stages of the activity (substitution, process change, innovation) as well as (e) advantages and disadvantages of digitalisation in context of the activities (e.g. financial and time-specific aspects, complexity, required expertise, transparency, data protection).

As a starting point, a few base publications were chosen to be scanned for relevant aspects regarding the research questions. From these notes and discussions, the coding tree was further developed and adapted after the first publications were coded. Advantages and disadvantages were developed inductively while doing the first base coding. The framework was developed further by using the basic principles of content analysis (Kuckartz 2018).

**Fig. 4:**
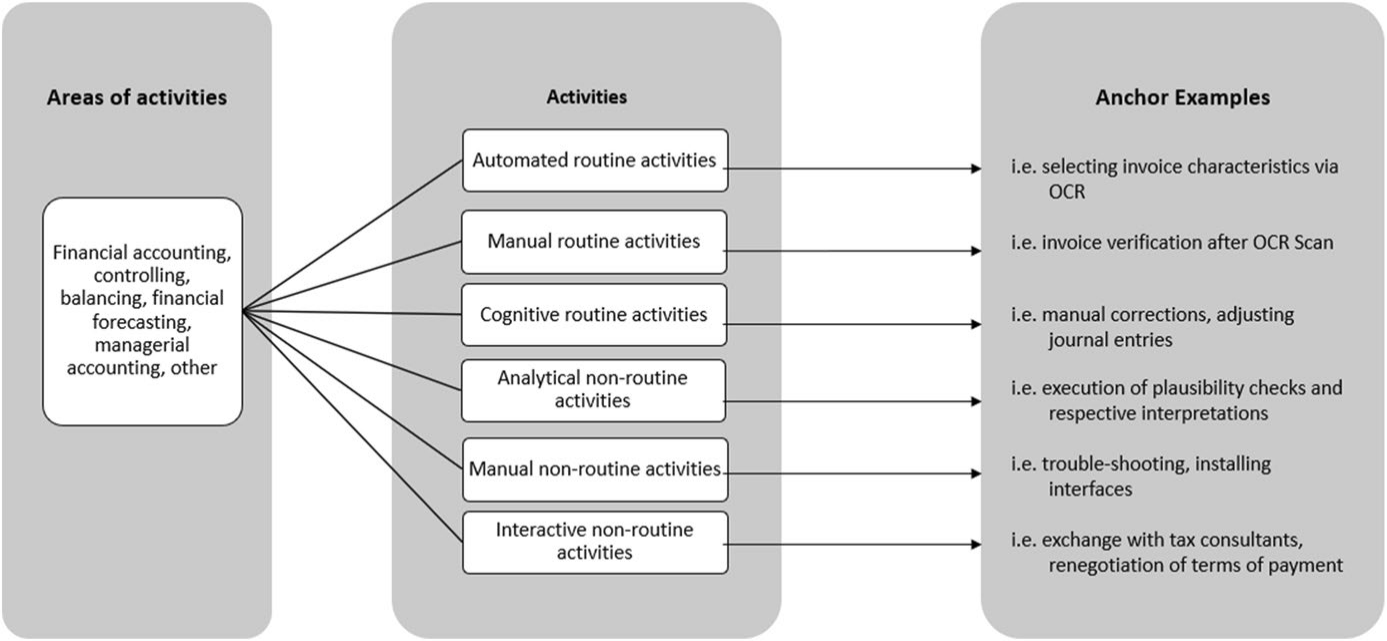
Overview of the coding manual for accounting activities

In the course of a special coding training, the final coding tree in the software MaxQDA was then distributed to two coding teams (three and two people each) along with the coding manual (Fig. 4). The sub-categories in the main category “Activities in Accounting” were further distinguished according to the aforementioned types of activities (Table 1), reaching from *automated routine* to *interactive non-routine activities* (Fig. 9). This more detailed structure allowed interpretations about the manners in which activities might have changed due to digitalisation.

To help the coding process, anchor examples were selected for all categories. As this paper is centred around activities, we are going to illustrate this process for the activity-related categories. For example, an *automatic activity* in accounting is characterised by a fully automated workflow like an automated deduction of worst-case scenarios with algorithms. A *manual routine task* does not require cognitive activation as its execution is part of the employee’s regular working routine, for example sorting accounting documents by date or verifying by hand if the positions on an invoice are complete and filled in correctly by the software. In contrast to this, a *cognitive routine activity* is constituted by a cognitive activation as it would be needed for manual corrections in bookkeeping or the adjustment of false journal entries. An *analytical non-routine task* could, for example, be the preparation and analysis of various financial statements, while *manual non-routine activities* encompass all tasks that are not part of everyday tasks, like solving technical problems or handling issues regarding hardware maintenance. Lastly, *interactive non-routine tasks* require a communicative element, like onboarding processes in the division or consultation with management. Thus, there are different levels of activities that need further specification and presumably undergo different changes through digitalisation.

To identify the extent of changes in the field, activities were assigned to a digitalisation stage. Hence, we applied the three aforementioned stages of digitalisation based on Bleiber (2019) and Hübl (2020) to identify the scope of changes in accounting: *substitution*, *process change,* and *innovation* (Fig. 1).

- Stage 1, substitution, is completed whenever analogue data or activities are exchanged for automated or digital ones. Neither process nor output is changed, however. This stage applies to activities using, for example, OCR or robotic process automation (RPA) or whenever the publication implies that a manual activity was exchanged for automation.
- In stage 2, process change, workflows, and processes are altered by the use of technology while the output of the process remains the same. This stage applies to all activities that use improved RPA technologies as well as process optimising or mining tools, such as partially automated bookkeeping.
- Stage 3, innovation, implies that both processes and their respective outputs are transformed through the use of technology, like artificial intelligence (AI), (deep) learning systems, machine learning, and neural networks. An example of this stage is a fully autonomous bookkeeping workflow.

## Results

### Bibliographic information and main foci of publications

**Fig. 5:**
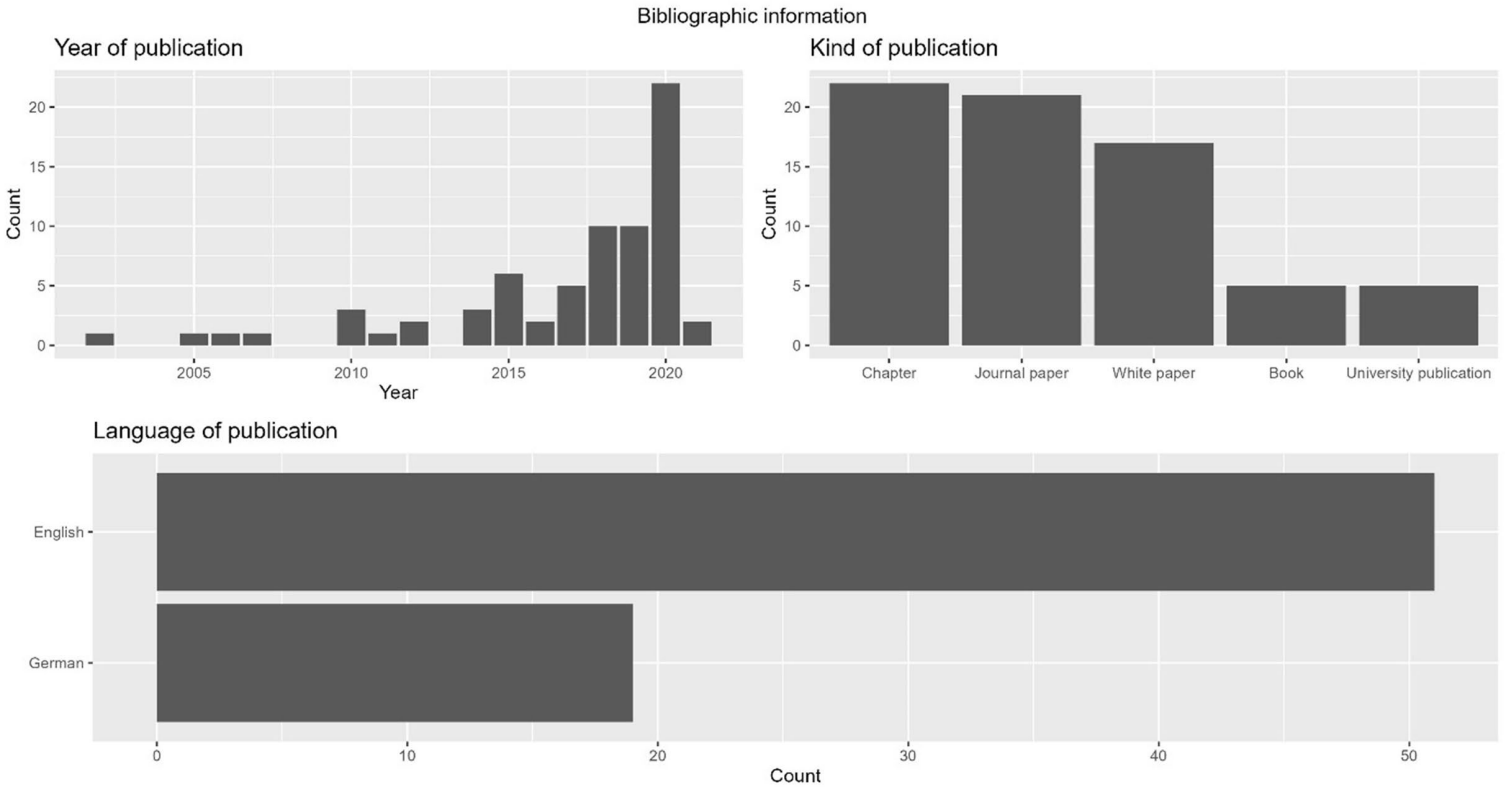
Bibliographic information (year of publication, kind of publication and language of publication

The majority of publications are in German (n = 51) and in English (n = 19). The dominance of German publications occurred due to the exclusion criteria. In our original set of 190 possibly relevant publications (Fig. 2), the distribution was fairly balanced with 98 publications in German and 92 in English. However, we excluded those publications which dealt with IT in a general manner (missing a direct link to accounting) and those which focused on other aspects of accounting but did not explicitly mention accounting activities. Most of these publications were in English, hence the large difference in the final data set. While most publications are either book chapters (n = 22) or journal articles (n = 21), whitepapers (n = 17), books (n = 5), and university publications (n = 5) constitute the minority. Looking at the year of publication, most sources were published between 2015 and 2020 (Fig. 5), fewer sources between 2000 and 2005. This rapid increase in publications indicates that digitalisation in accounting has gained popularity within the past five years and is now a leading topic in accounting publications.

**Fig. 6:**
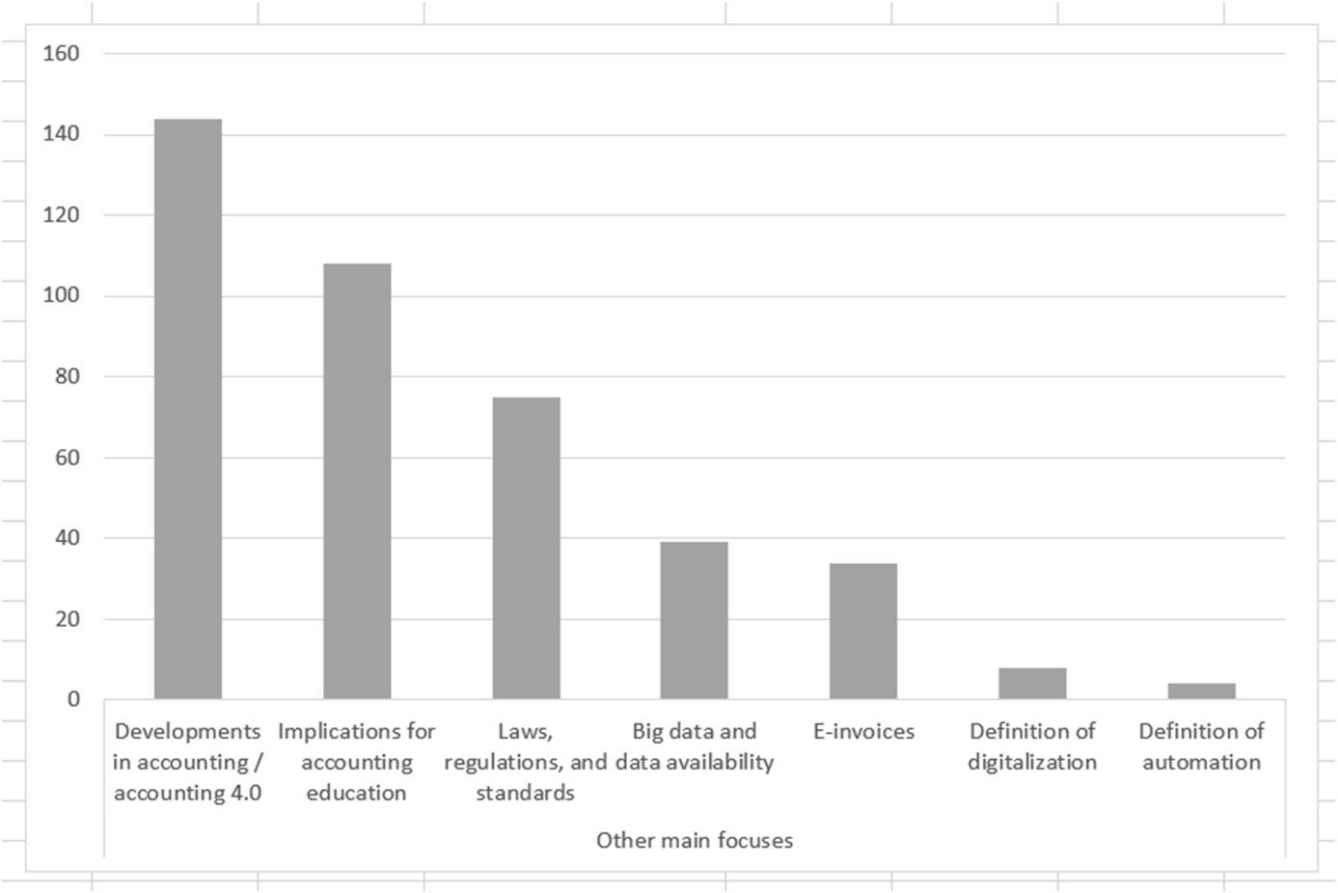
Main foci of publications (number of codes, multiple codes per paper possible)

The sub-categories in “main focus” could be coded multiple times within the same publication. Besides the technologies and software systems in use, the two most frequently mentioned aspects regarding the main foci are *developments in accounting/accounting 4.0* (n = 144) and *implications for accounting education* (n = 108), a category that describes possible changes in the configuration of activities. (Fig. 6). These two categories are future-oriented and involve both the (technical) developments and the industry’s dynamic requirements for accounting graduates and implications for education.

Regarding the *developments in accounting*, authors primarily describe processes that are improved and standardised to maximise productivity and the accountant’s value to the company (Baier 2019; Müller and Reichmann 2010). Especially technologies like advanced analytics and RPA are becoming increasingly important in accounting 4.0 (Egle et al. 2020; Koch 2017; Losbichler and Gänßlen 2018; Satzger et al. 2018).

Special aspects that were not mentioned in an educational context, such as the related legal requirements (n = 75) or the generally increasing data availability (n = 39), are shown in separate categories. Several publications also deal with the concrete implementation and procedure of electronic invoices (n = 34). The categories *definition of automation* (n = 4) and *definition of digitalisation* (n = 8) are mentioned the least frequently.

### RQ 1: Activities concerned with digitalisation in the different areas of accounting

#### Areas and types of activities in accounting

**Fig. 7:**
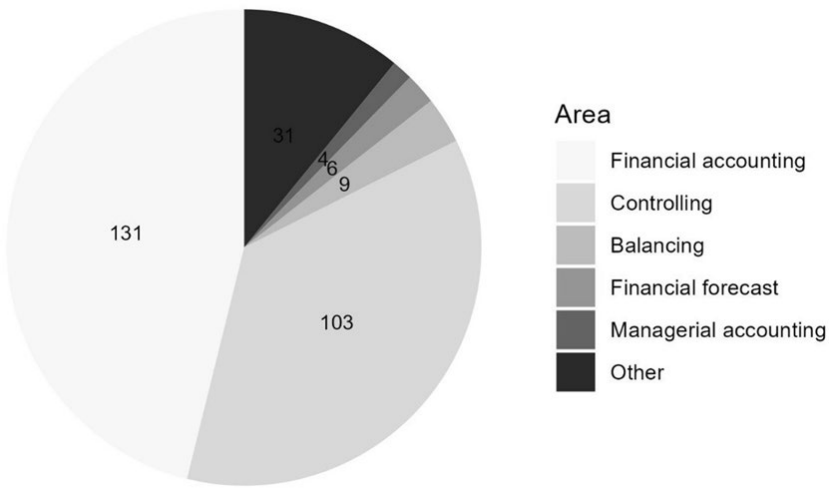
Areas of activities in accounting divisions

**Fig. 8:**
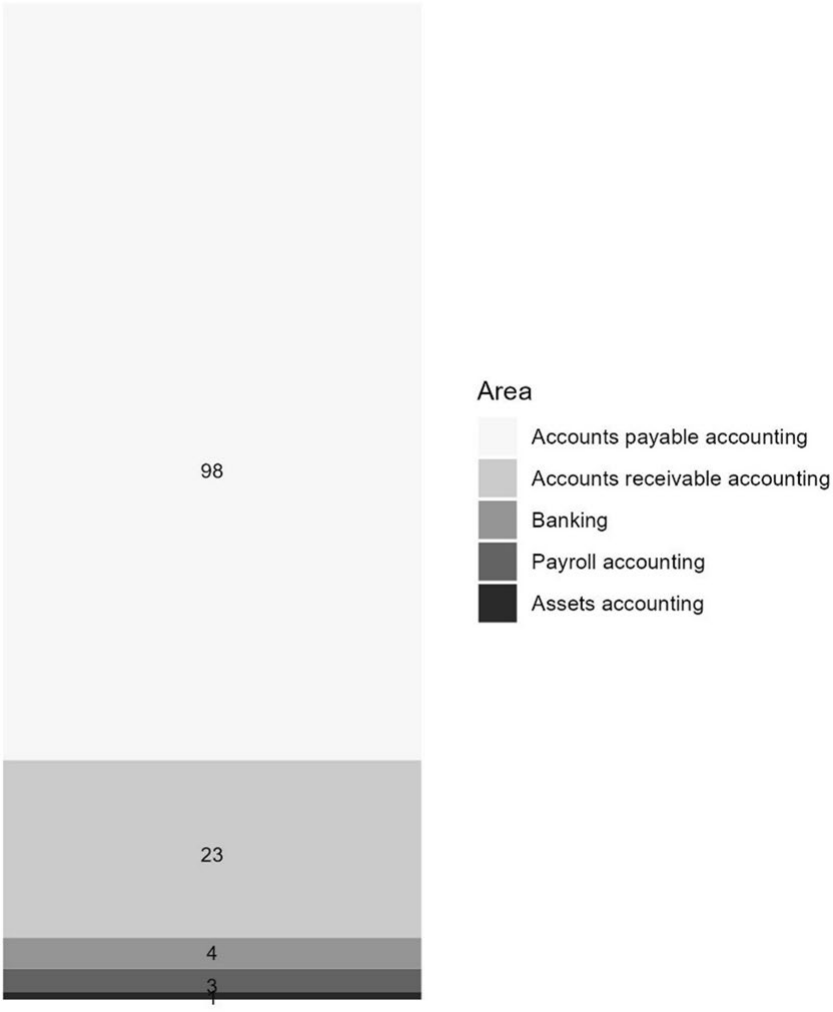
Areas of activities in financial accounting sub-divisions

A total of n = 285 activities are distributed across six accounting areas including “other” activities (Fig. 7). Most frequently mentioned are activities concerning financial accounting (n = 131) and controlling (n = 103). In contrast, all other divisions together make up roughly 25% of the activities described in the publications. The financial accounting code is given to all activities that are rooted in the accounts payable, accounts receivable, banking, payroll, and asset accounting areas (Fig. 8). The activities in the area of financial accounting are dominated by *accounts payable accounting* (n = 98). Many publications focus on e-invoicing, a technology employed to increase productivity in both accounts payable and *accounts receivable accounting*, the second most mentioned sub-division (n = 23). In addition, OCR and RPA technologies are often used to increase efficiency in both divisions.

The second most frequently mentioned category regarding activities, controlling, mainly encompasses aspects such as reporting, communication, and interpretation of data (Bär et al. 2019; Egle and Keimer 2017; Heupel and Lange 2019). In contrast to financial accounting, the category of controlling does not focus as much on technologies but rather on specific changes in the job profile (Becker et al. 2020; Losbichler and Gänßlen 2018; Schindera et al. 2018).

**Fig. 9:**
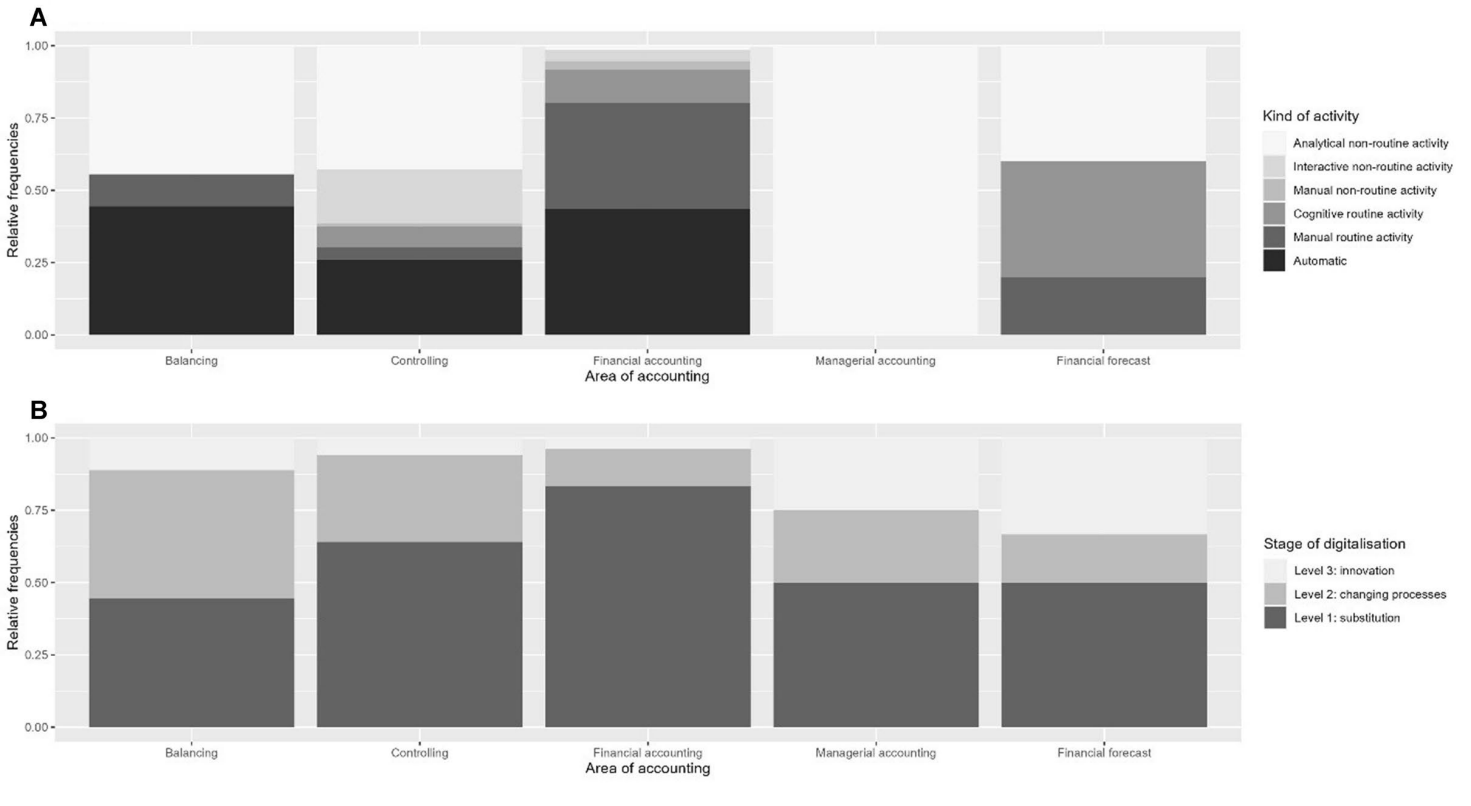

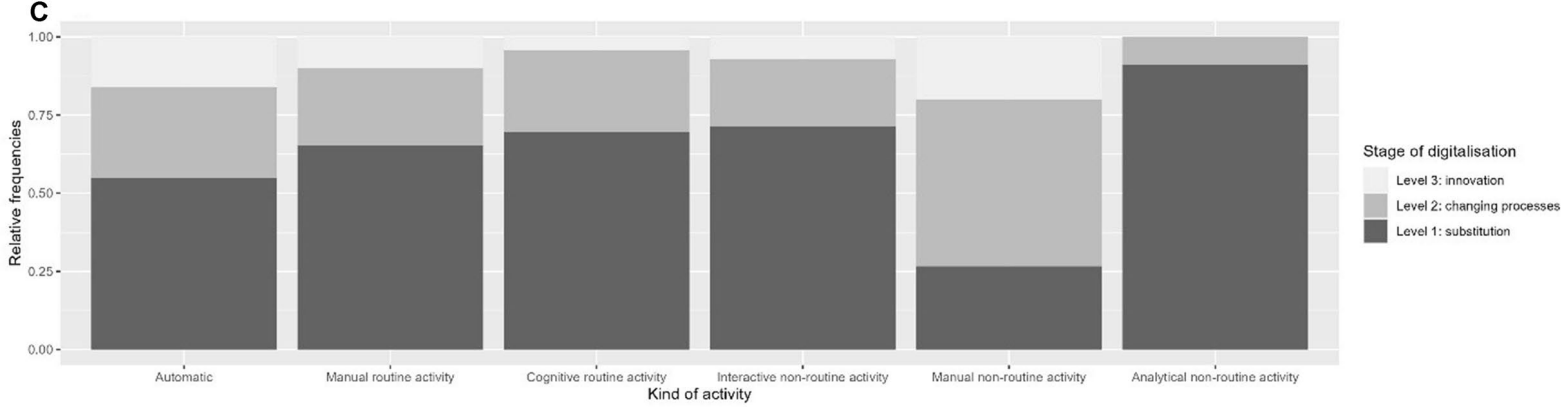
**A**–**C** Types of activities in different accounting areas

The types of activities in the different areas of accounting (Fig. 9A) are differentiated by their mean of action. More specifically, Fig. 9A describes what kinds of activities typically occur in the different areas of accounting according to our analyses. In Fig. 9, those numbers are given as relative numbers because a publication had sometimes multiple codes of the same activity. To account for these duplicates and to maintain proportions, the absolute frequencies are divided by the respective totals per sub-category. As an example, the category *financial accounting* is mentioned 131 times of which 58 mentions are for automatic activities. Thus, there are *automatic activities* without manual dimensions and others that are *routine activities* (manual and cognitive) and *non-routine activities* (manual, interactive and analytical).

In the area of financial accounting, most reported activities are either automatic or manual routine activities. Automatic activities are mentioned frequently, for example the automatic recognition of invoices via RPA or their generation directly from the ERP system, although a fully automated process is not common practice yet (Appelfeller and Feldmann 2018; Jordanski 2020; Kreher 2021; Schömburg and Breitner 2010; Tanner 2016). Instead, some parts of the process are automated and some remain manual. This explains the ratio of manual routine activities, as publications often address the manual correction or computation of invoices (Bernius and Kreuzer 2014; Koch 2017; Menges 2012; Wilczek 2014). Other areas of accounting like *balancing* or *controlling* display a similar amount of automatic activities that primarily include the use of AI in balancing (Kink 2007; Le Guyader 2020). Another aspect is the automated detection of variances and automated reporting through machine learning in controlling (Alexander et al. 2019; Ashoka et al. 2019; Jonen 2020). In contrast to publications that focus on financial accounting, authors who address managerial accounting topics identify a high proportion of analytical non-routine activities that primarily include the use of RPA to distribute reports and allocate resources which then have to be analysed by human employees (Langmann and Turi 2020).

### RQ 2: Accounting activities in different stages of digitalisation

The codes regarding areas of activities in accounting alone, however, do not provide any information regarding the quality of the activities or the respective stages of digitalisation that were presented earlier in this paper. To formulate statements about the quality of accounting activities, it is necessary to analyse the stage of digitalisation and the respective frequencies in publications (Fig. 9B–C).

### Stages of digitalisation in the different areas of accounting

Regarding the stages of digitalisation, the first stage (substitution) is assigned the most across all accounting divisions (Fig. 9B). Especially activities that remain in the first stage of substitution are attributed to financial accounting. In contrast to this area of accounting, both financial forecasting and managerial accounting areas show a larger proportion of digitalisation that has progressed to stages 2 and 3, process change and innovation. As to managerial accounting, this extends to the integration of a controlling software that introduces innovations in processes in the respective divisions and the implementation of new processes (Kink 2007; Raschig and Schulze 2020; Selb 2020). In financial forecasting, most processes are still in the substitution stage (Klein and Küst 2020), but some have progressed to integrate process change or innovation, such as the software-based analysis of reporting data for a proactive controlling approach as shorter product lifecycles necessitate shorter planning horizons (Kink 2007; Sledgianowski et al. 2017).

### Stages of digitalisation in the different activities

In extent to the types of activities (Fig. 9C), most *routine tasks* (see Table 1 for these categories) are still mainly in the substitution stage. For instance, RPA technologies are used regularly to substitute manual with automated processes (Gadatsch 2020) or electronic invoicing is integrated (Jordanski 2020; Klein and Küst 2020; Pagel 2019; Schömburg and Breitner 2010). Approximately one third of automated activities are in the stage 2 of process change, for example when the invoicing process is adapted to a digital format for both incoming and outgoing transactions (Kreher 2021).

For *non-routine activities*, the trend is mostly different. Interactive non-routine activities consist of a larger proportion of stage 2 digitalisation. Those process changes established in the publications include vendor inquiries through automated online portals (Binkow 2015) or the supervision of RPA systems or AI algorithms (Hmyzo and Muzzu 2020; Klein and Küst 2020). Processes need to be standardised to allocate controllers more resources to handle more detailed analyses, interpretations, and communications of results and to minimise their routine activities (Keimer and Egle 2020). Schindera et al. (2018) express that, while the chief financial officer slowly transforms into a chief performance officer, employees in the controlling division are confronted with a changing of roles, too—into that of a business partner. This change is conditioned by the use of big data analytics. Thus, analytical non-routine activities gain in importance. Across the areas of accounting, there is a connection between the kind of activity and its respective stage of digitalisation that has not yet been quantified. While the majority of automatic and routine activities remain in stage 1 digitalisation, *interactive non-routine activities* predominantly are assigned to stage 2. This finding can be explained by the successful implementation of software solutions and technologies such as AI leading to more activities involving it in the second stage (Chen et al. 2012; Heupel and Lange 2019; Le Guyader 2020; Losbichler and Gänßlen 2018).

*Analytical non-routine activities,* however, show a contrasting result with most activities in stage 1 as employees need to make manual corrections (Jordanski 2020). Thus, a human component is required in most processes. However, technologies such as blockchain or AI provide the chance to increase the stage of digitalisation in the future (Chen et al. 2012; Grönke and Heimel 2015; Trachsel and Bitterli 2020). In contrast to manual routine activities (e.g. e-invoicing), the integration of software solutions for analytical non-routine activities requires more resources, hence the lower threshold (Arbeitskreis Externe Unternehmensrechnung der Schmalenbach-Gesellschaft für Betriebswirtschaft e. V. 2018; Egle et al. 2020; Pagel 2019; Weiber et al. 2002).

### RQ 3: Placement of activities within the supply chain

**Fig. 10:**
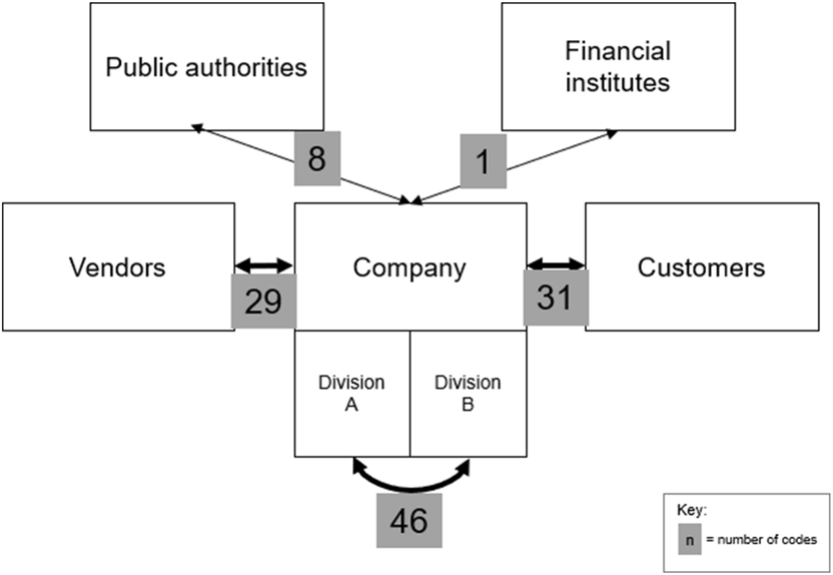
Interfaces (placement of activities within the supply chain) addressed in the publications

Different types of the described activities in different accounting areas can be established and interlinked within a company with the aid of interfaces (Fig. 10). Within a single company, there can be a multitude of interfaces across divisions and along the supply chain. These interfaces are often connected to the activities, for example internal and external financial accounting, which process inbound and outbound invoices or to the procurement division that interacts with vendors. Most papers address one or more relevant interfaces. Most frequently mentioned are *internal interfaces* (n = 46), especially regarding the adaptation of accounting and controlling division structures to new business processes or ERP implementation (Binkow 2015; Gadatsch 2020; Heupel and Lange 2019; Najderek 2020; Suden 2010). The rising focus on process management to increase productivity is depicted in the interfaces; more extensive cooperation between financial divisions due to cross-sectional processes gains in importance (Arbeitskreis Externe Unternehmensrechnung der Schmalenbach-Gesellschaft für Betriebswirtschaft e. V. 2018; Bayerl et al. 2020). A functioning flow of information and close cooperation between divisions can aid the success of digitalisation projects. Implementing standardised software across the company is identified as a suitable approach to minimise interface issues (Becker et al. 2020; Gadatsch 2020; Hecht and Scherrer 2020).

Interfaces with *vendors* (n = 29) or *customers* (n = 31) are also mentioned. The main context of vendor interfaces lies in the dimension of electronic invoicing and the conditions for successful implementation as well as specific processes (Appelfeller and Feldmann 2018; Klein and Küst 2020; Menges 2012; Najderek 2020; Pagel 2019; Suden 2010; Tanner 2016). It is necessary to harmonise the vendor’s system requirements with the company’s system requirements (Tanner 2016). Sometimes, companies establish a supplier self-service that enables the vendors to manage processes such as invoicing or the placement of orders within the company’s interface (Appelfeller and Feldmann 2018). At the same time, interfaces with customers are mostly focused on improving their experience by distributing invoices digitally or implementing customer self-service (e.g. checkout via smartphone), thus optimising the company’s commodity, liquidity, or information flows (Appelfeller and Feldmann 2018; Binkow 2015; Cong et al. 2018; Egle and Keimer 2017; Jonen 2020; Klein and Küst 2020; Nagel 2018).

### RQ 4: Technologies used to perform the accounting activities

**Fig. 11:**
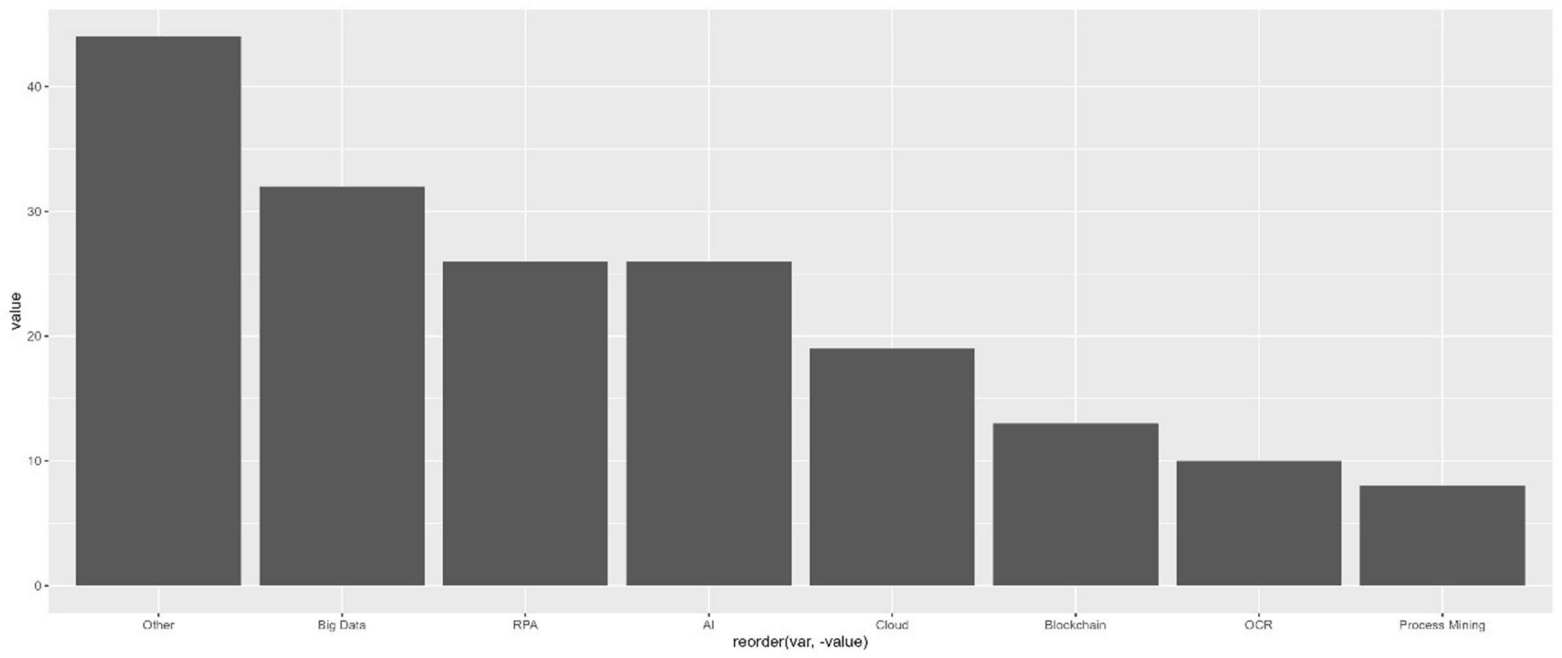
Frequency of mentioned technologies

Most publications address one or more technologies in the context of accounting (Fig. 11). The specific technologies mentioned most frequently are big data analytics (n = 32), RPA (n = 26) and AI (n = 26). Process mining, which allows companies to analyse their processes through digital technology, is mentioned least frequently (n = 8). This result supports the perception of those technologies as the main disruptors in the industry. *Other technologies* are mentioned in 44 publications. These are mostly generalised statements that refer to basic technologies and software like ERP systems or technology that can be used to process e-invoices (Gadatsch 2020; Koch 2017; Schindera et al. 2018; Schömburg and Breitner 2010; Tanner and Wölfle 2005; Wilczek 2014).

Ashoka et al. (2019) suggest that the use of AI in accounting will free up employees’ time capacities and reduce repetitive activities. They also predict a shift towards internet-based reporting. Egle and Keimer (2017) add that the number of repetitive activities will likely be reduced through AI and big data analytic tools, primarily by minimising the number of manual decisions accountants and auditors have to make in their routine activities. These internal factors are supplemented by external aspects that address the customer journey. Especially, AI is used to improve the customer experience (Appelfeller and Feldmann 2018; Bayerl et al. 2020; Egle et al. 2020). In addition, big data is often mentioned in the context of customers (Gadatsch 2020; Losbichler and Gänßlen 2018; Schindera et al. 2018). Yet, certain advantages for other areas of accounting are made visible as analysis tools may aid, for example, auditors in data management or controllers in forecasting (Pan and Seow 2016). This may be the first indication of a shift in the scope of activities in accounting and controlling towards increasing digitalisation.

Especially, repetitive and physical activities such as monthly reporting or e-invoicing profit from the implementation of RPA (Bowles et al. 2020; Sandner et al. 2020). However, controlling and accounting divisions can be further improved, too (Bayerl et al. 2020; Koch 2017; Langmann and Turi 2020). Through RPA solutions, up to 50% of all back-office tasks—like the synchronisation of supplier and customer accounts—could be automated in the upcoming years (Koch 2017). Additionally, the number of employees necessary to manage the workload can be reduced or shifted to new tasks (Kirchberg 2017). Up to 36% of companies already use RPA or AI for individual tasks in their accounting department (Kreher 2021).

### RQ 5: The advantages and disadvantages of digitalised accounting activities

**Fig. 12:**
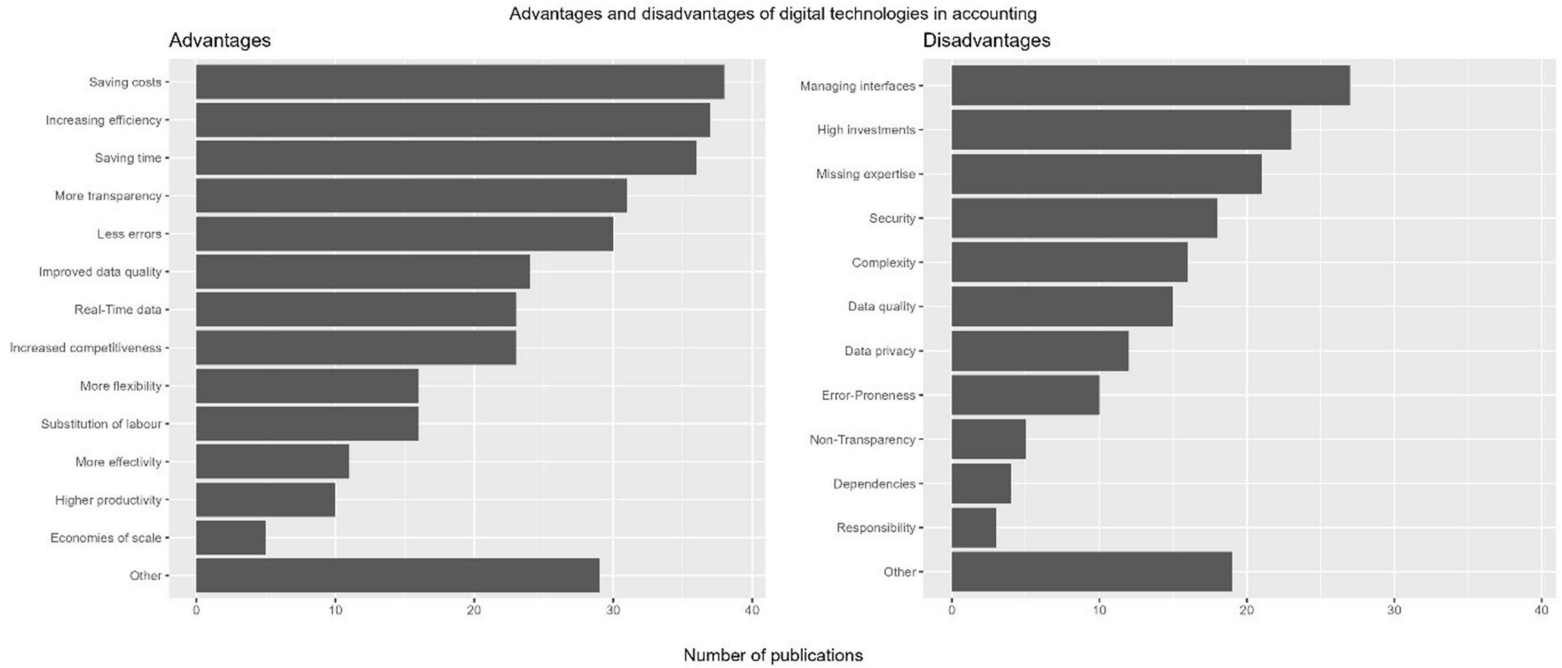
Advantages and disadvantages of digitalisation in accounting (number of publications)

In the review, a variety of advantages and disadvantages of digital technologies in accounting became apparent (Fig. 12). In total, we found almost three times as many advantages (n = 618) named than disadvantages (n = 249).

The advantages mentioned most frequently were *saving costs* (n = 90), *increasing efficiency* (n = 76), and *saving time* (n = 73). One example is that digital tools help accountants to manage their workload better and at lower costs (Pan and Seow 2016). Keimer and Egle (2020) state that technologies such as big data analytics or cloud solutions are useful to reduce the susceptibility to errors while simultaneously increasing efficiency in the controlling division. In addition, process automation is perceived as a key aspect of efficiency enhancement in the auditing sector (Werner and Gehrke 2011). The advantage of *saving time* is connected to two time-related opportunities. Firstly, Pagel (2019) mentions the aspect of processes optimised by digital technologies; lead time for invoices was reduced by up to 15 days, leveraging liquidity on the biller’s side. The freed-up worktime indicates the second perceived opportunity: The specialised workforce in the controlling division can shift their responsibilities to less administrative tasks, thus extending current activities to strategy-oriented areas (Egle and Keimer 2017).

These productivity-related factors are mentioned in half of the publications. The advantages of *economies of scale* (n = 7) and *productivity* (n = 11) are only rarely mentioned. For example, Egle and Keimer (2017) discuss *productivity* as an advantage that results from saved time and error reduction.

“Other” advantages (n = 57) range from employee engagement and motivation (Langmann and Turi 2020) to simplified auditing processes (Sledgianowski et al. 2017) or improved understanding of financial events (Ashoka et al. 2019). But customer satisfaction (Binkow 2015) and sustainability-driven aspects such as CO_2_ reductions (Bernius and Kreuzer 2014) were mentioned as additional aspects as well.

Regarding the disadvantages, content analysis shows the highest risks with digital technologies in accounting in the areas of *interface management* (n = 40), *financial investments* (n = 42), and *missing expertise* (n = 31), mainly with new technologies (Fig. 12). Gadatsch (2020) appoints the central issue of interface management to the complex implementation and its extensive time frame. In addition, data redundancies created by the parallel upkeeping of former and new ERP systems might further increase interface issues, effectively increasing the workload. Thus, there is a need for technological and methodological skills for successful interface management. Schömburg and Breitner’s (2010) explanations support this result. They identify the main reasons for companies to defer process digitalisation as a lack of expertise, necessary investments, and implementing suitable software. This finding primarily connects to electronic invoicing (Jordanski 2020; Koch 2017; Nagel 2018; Najderek 2020; Schömburg and Breitner 2010; Tanner 2016; Tanner and Wölfle 2005) and ERP-specific areas (Appelfeller and Feldmann 2018; Binkow 2015; Chen et al. 2012; Cong et al. 2018; Fuller and Markelevich 2020; Gadatsch 2020; Jonen 2020; Wilczek 2014). The “other” disadvantages (n = 32) focus on the risks of decentralised innovations that are not implemented company-wide (Schindera et al. 2018) as well as the amount of time needed for software maintenance or to train employees (Gadatsch 2020). In addition, a few technology-specific disadvantages are mentioned: On the one hand, the implementation of RPA does not require the innovation of new processes, thus old, ineffective processes could stall future changes (Gadatsch 2020). On the other hand, blockchain could hinder scalability due to slow transaction speeds and challenging error corrections (Fuller and Markelevich 2020).

## Discussion and summary

### Summary

**Fig. 13:**
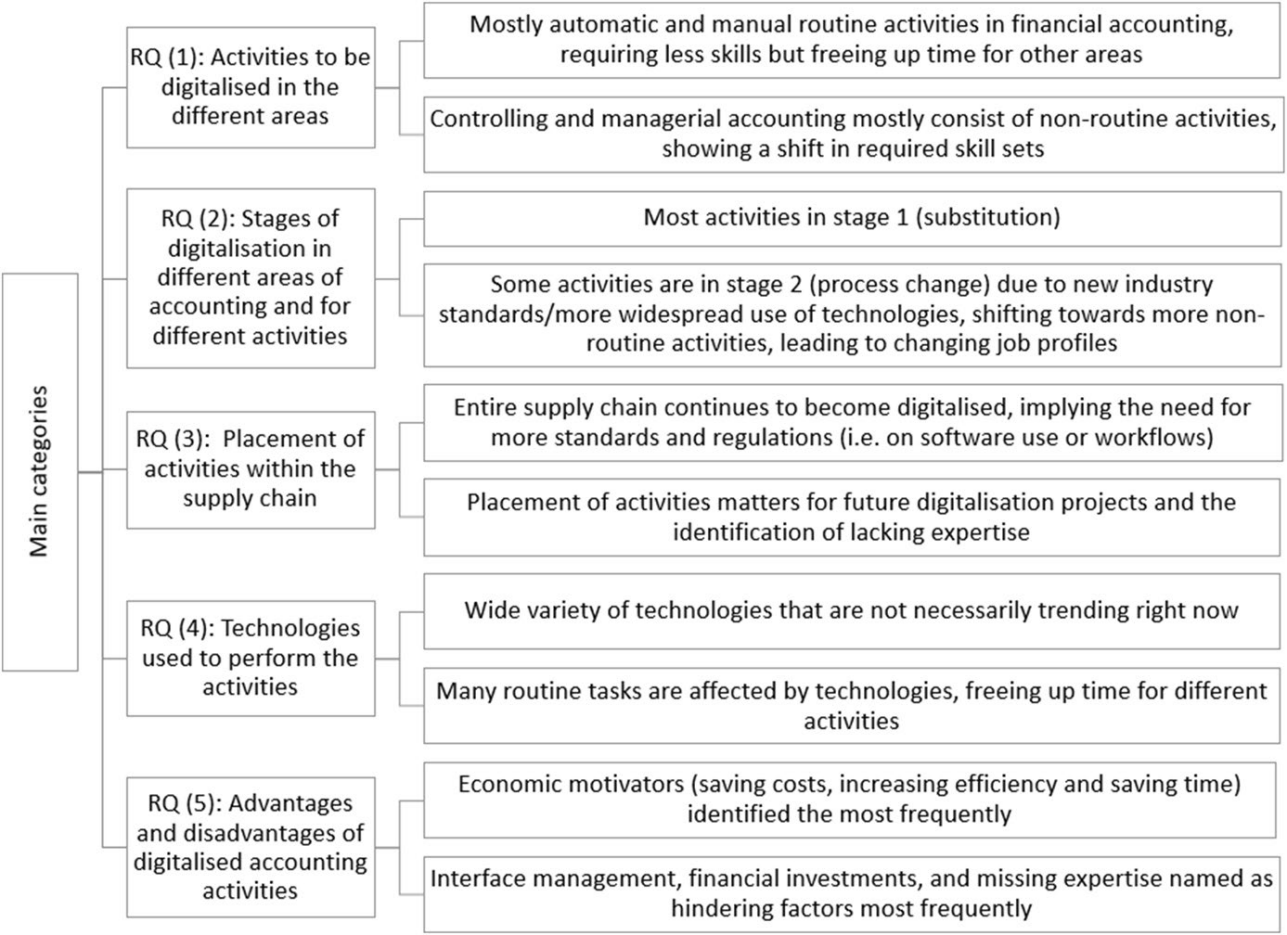
Overview of findings

Digitalisation is changing numerous processes in the working lives of accounting professionals. This also applies to processes associated with accounting. Against the background of the question of how accounting graduates will have to be trained in the future, we have identified requirements and activities with the help of a systematic literature review. The literature review was conducted based on a conceptual framework of six types of activities and three stages of digitalisation. Figure 13 gives a condensed overview of the most important findings:

Regarding the foci of publications dealing with digitalisation in the field of accounting, we found that developments in accounting and implications for accounting education dominate. These findings indicate that the discourse in the field has moved beyond the definitory stages and towards the identification of needs of action, including, among others, the advancement of accounting education and adjustments of curricula.

Results for RQ (1) which deals with activities in the areas of accounting that are subject to digitalisation hint at financial accounting and controlling divisions being the main areas of change. While in financial accounting, there is a focus on technology-based change, the area of controlling mostly encompasses changes in the controller’s job profile that are ensuing due to the increasing digitalisation of current tasks. As most activities in financial accounting are based in the area of accounts payable, the focus on technologies could be connected to efficiency-related opportunities that technological updates may implicate. Out of the six types of activities, analytical non-routine, automatic, and manual routine activities are the predominant categories (see Table 1 for an overview of definitions). This finding can be linked to the different degrees of automation in the different areas: In financial accounting and balancing, a large share of tasks has been automated, for example by RPA. In controlling, financial forecasting, and managerial accounting, however, the leading tasks are data analysis (cognitive routine) and consultancy (analytical and interactive non-routine). These results also provide first hints at the stages of digitalisation in the different areas of accounting (RQ 2).

In RQ (2), we identified different stages of digitalisation in the different areas. The results show that the proportion of digitalised activities is largely dependent on the area, yet most activities currently remain in the substitution stage. This indicates that the integration of technologies and process changes is still underway. The combined analysis of areas, activities, and stages was particularly revealing because it allowed differentiated interpretations. The analysis showed, among other things, that types of activities and different accounting areas are digitalised at different stages. Stage 1 (substitution) is to be expected, for example, in financial accounting and analytical non-routine activities. In contrast, interactive non-routine activities can be found more often in the second stage of digitalisation because current processes are supplemented by new technologies. This finding matches the results from a study by Sachs et al (2016) who focused on changing job descriptions in the commercial field. Interaction is required to communicate results and consult with management. This leads to expanding job profiles and further required competences that trace back to updates in accounting education.

All the activities we identified require different skill sets and are placed at different interfaces along the entire supply chain (RQ 3). This implies the supply chain’s continuous digitalisation requiring new technical standards and agreements. For example, the increasing popularity of e-invoicing has motivated efforts regarding the standardisation of software and workflows.

Another aspect that shifts competence requirements is the implementation of major technologic trends such as AI and big data analytics in the industry (RQ 4). Frequently mentioned technologies and corresponding business processes need to be integrated into accounting curricula to meet industry needs. On the one hand, technology itself and its evaluation could be addressed. On the other hand, the handling of technology could be prepared when it is integrated into industry practice. Learning materials need to reflect these changes and thus become more digitalised as well.

With RQ (5), we analysed the advantages and disadvantages that the publications under analysis identified during the process of digitalisation in the field of accounting. Even if the advantages mentioned in the publications, such as efficiency reasons, outweighed the disadvantages, risks such as interface management and accounting professionals’ lack of skills and competences must still be considered. In consequence, companies lack the expertise to successfully manage digital transformation processes, underlining the importance of specific competence development. Other factors that are main motivators are of a classic economic nature, such as an increase in overall productivity by saving time and financial resources. Both technologies’ specific disadvantages and the process of digitalisation in accounting more generally suggest the need for improved accounting graduate education and further training or at least for more guidance for both employers and employees. This need applies to concise abilities, such as the assessment of digital technologies and processes, and the criteria-based evaluation of the potential of software implementation. Depending on how quickly corresponding competences can be developed in the company’s workforce, flexibility in competence acquisition for varying technologies and the successful implementation as well as maintenance of technological interfaces is needed. Thus, the field of accounting has reached a level of digitalisation that entails changes in competence profiles to enable both accounting professionals and graduates to manage new demands.

### Limitations

#### Methodological limitations

Concerning limitations, our paper focuses primarily on results from a literature review and not on the analysis of real company situations or accounting professionals’ reports. The activities, stages, and combinations described in the articles were evaluated according to the number of codes. Whenever a publication mentioned the same code in a different context, it was coded as a new mention. This could potentially distort results. In addition, we did not analyse the extent to which technologies have actually been established in companies and in particularly in accounting. The papers might reflect the past and current states of the discussion.

The next limitations stem from the selection process of publications and their respective language of publication. While there were specific journals in English selected as relevant, we did not select specific German journals. Regarding the dominance of German-speaking publications, it is possible that the databases we used, particularly Google Scholar, produced a bias due to their search algorithms that might include geolocation. To balance this out, we searched for our keywords in both English and German. However, we cannot ignore that this might have implications for the results of our study. In German-speaking countries, accounting methods are rather defensive compared to English-speaking countries, where future-oriented perspectives are more common. In consequence, it is possible that some of our results only pertain to Germany and other German-speaking countries as well as to some to other countries, especially those results that are connected to the flexibility and design of accounting curricula or the stage of digitalisation in the industry.

### Content-related limitations

Another limitation is the focus on controlling and financial accounting in the keyword groups as other areas of accounting might have been underrepresented. As for the integration of digitalisation into accounting curricula and learning materials, our paper does not consider external factors that might influence the creation and adaptation of accounting curricula like accountancy professional bodies and other accrediting organisations.

### Implications for accounting education and professional practice

The lack of expertise takes a prominent position in our analysis, which is mainly reflected by the prevention of digitalisation processes and innovation of accounting activities. Particularly, technological and methodological skills are needed to integrate new technologies, innovate processes, and, in consequence, produce a shift in activities. The following key implications can be deducted for accounting education and professional practice (see for more details Berding et al. 2022):

- Adaptation of curricula to reflect the change of focus from financial accounting and routine activities to the area of controlling and non-routine activities ("RQ 1: Activities concerned with digitalisation in the different areas of accounting" and "RQ 2: Accounting activities in different stages of digitalisation" section).
- Intensification of the use of technological and analytical tools in accounting education curricula to motivate early contact and contribute to the holistic understanding of processes as the importance of independent reporting and interpretation of financial key figures increases ("RQ 1: Activities concerned with digitalisation in the different areas of accounting" and "RQ 4: Technologies used to perform the accounting activities" section).
- Combined focus on technical and professional competences in the different areas of accounting that recognises the increasing importance of meta-competences such as proactive thinking, self-control, creativity, and interdisciplinary action. This is particularly important to ensure that graduates and employees stay capable to navigate the field of accounting and adapt to the changing parameters flexibly ("RQ 2: Accounting activities in different stages of digitalisation" section).
- Integration of data and process management and new technologies into compulsory curricula and professional development courses to generate a holistic understanding of processes and manage change across the entire supply chain ("RQ 3: Placement of activities within the supply chain" and "RQ 4: Technologies used to perform the accounting activities" section).
- Employees in the areas of accounting and controlling need improved communicative and entrepreneurial skills to market the added value of digitalisation to companies ("RQ 5: The advantages and disadvantages of digitalised accounting activities" section).

Overall, the analysis provides valuable hints for future-oriented accounting education that supports companies in realising a maximum of expertise to manage their digital transformation. This extends to the opportunity to think about implications for professional practices (How digitalised can the field be and how can digitalisation change job profiles?), for accounting students/employees (Am I willing to engage with changing technologies to improve my skill set and potentially work on entirely different activities within the next few years?), and even for society (How can these changes and their implications on competences help in finding creative solutions to 21^st^-century challenges?). This review can serve as a starting point for these and similar considerations.

This paper was intended to contribute to the discussion of future (competence) requirements for graduates in accounting. The detailed analyses provide an in-depth insight into six types of activities and the different stages of digitalisation for specific areas of accounting. This systematic and detailed analysis was necessary to gain knowledge on how to initiate concrete improvements in the content and methodology of the education and training of accountants (see for example Author 1 et al. 2022). It became clear, for example, that a deeper look into these areas is valuable as the same technologies are not likely to prevail in all areas at the same stage and for the same types of activities. In the area of financial accounting, for example, the duration of tasks decreases due to the large proportion of routine activities that can be automated or substituted, freeing up time for other activities or areas of accounting. One of these is controlling, where there are more non-routine activities that require new skills. However, it also became clear that interface management in particular is essential for linking the numerous technologies. Against this background, the didactics of accounting are also challenged to educate and train graduates in a differentiated manner.

## Data Availability

The datasets used and/or analysed during the current study are available from the corresponding author on reasonable request.

## Appendix I: List of coded publications

**Table:**

Author	Year	Title and publication information	Type	Language
Alexander S, Tiefenbeck F, Sabirzyanova N	2019	Digitale Transformation des Performance Managements: Zielbild und aktuelle Initiativen. [Digital transformation of performance management. Goals and current initiatives]. CON 31 (6): 39–42. https://doi.org/10.15358/0935-0381-2019-6-39	Journal Paper	German
Andiola LM, Masters E, Norman C	2020	Integrating technology and data analytic skills into the accounting curriculum: Accounting department leaders’ experiences and insights. Journal of Accounting Education 50:1–18. https://doi.org/10.1016/j.jaccedu.2020.100655	Journal Paper	English
Appelfeller W, Feldmann C	2018	Stufenweise Transformation der Elemente des digitalen Unternehmens. [Gradual transformation of the elements of a digital company]. In: Appelfeller W, Feldmann C (eds) Die digitale Transformation des Unternehmens: Systematischer Leitfaden mit zehn Elementen zur Strukturierung und Reifegradmessung. Springer, Berlin, pp 19–192	Book	German
Arbeitskreis Externe Unternehmensrechnung der Schmalenbach-Gesellschaft für Betriebswirtschaft e. V	2018	Chancen und Herausforderungen der Digitalisierung für die Effektivität und Effizienz des Rechnungswesens. [Opportunities and challenges of digitalisation for the efficiency and efficacy of accounting]. In: Krause S, Pellens B (eds) Betriebswirtschaftliche Implikationen der digitalen Transformation. Springer Gabler, Wiesbaden, pp 301–17. https://doi.org/10.1007/978-3-658-18751-4_16	Chapter	German
Ashoka, ML, Abhishek N, Divyashree MS	2019	Emerging Trends in Accounting: An Analysis of Impact of Robotics in Accounting, Reporting and Auditing of Business and Financial Information*.* International Journal of Business Analytics & Intelligence 7(2): 28–34	Journal Paper	English
Baier T	2019	Digitalisation in Management Accounting. Bachelor thesis. Berlin: Hochschule für Wirtschaft und Recht. Available via https://www.theseus.fi/handle/10024/262141. Accessed 21 Mar 2023	University Publication	English
Bär J, Badura D, Bockshecker A, Hauer L, Karalash M, Nehls S, Neuhaus U, Schröder H, Schulz M, Sharma V, Welter F	2019	Die Mitarbeiter von Morgen: Ergebnisse eines Workshops zu den Kompetenzen künftiger Mitarbeiter im Bereich Business Analytics. [The future's employees: results from a workshop about the skills of future employees in Business Analytics]. Nordblick – Hochschule der Wirtschaft (8):34–49	Whitepaper	German
Bayerl E, Krippner K, Sikora C	2020	Steuerrechtliche Anforderungen und Entwicklungen im Bereich Steuern und Rechnungswesen [Tax law requirements and developments in the scope of taxation and accounting]. In: Rosar W, Krippner K, Setnicka M (eds) Digitalisierung Im Steuer- Und Rechnungswesen. Linde, Wien, pp 277–401	Chapter	German
Becker W, Nolte M, Schuhknecht F	2020	Die Rolle des Chief Financial Officer im Rahmen der digitalen Transformation von Geschäftsmodellen. [The role of the chief financial officier in the scope of the digital transformation of business models]. In: Keimer I, Egle U (eds) Die Digitalisierung Der Controlling-Funktion: Anwendungsbeispiele Aus Theorie Und Praxis. Springer Gabler, Wiesbaden, pp 373–400	Chapter	German
Bernius S, Kreuzer S	2014	Warum eRechnung? Ökonomische und ökologische Einsparpotenziale in der öffentlichen Verwaltung. [Why e-invoicing? Economic and ecological saving potentials in public administration]. In: Rogall-Grothe C (ed) Leitfaden Elektronische Rechnung in der öffentlichen Verwaltung: Grundlagen, Umsetzungsempfehlungen, Best Practices. Goethe Universität Frankfurt (Main), pp 33–42	Chapter	German
Bernius S, Pfaff D	2014	Mythen der eRechnung – Wie wissenschaftliche Erkenntnisse den Weg zur Umsetzung des elektronischen Rechnungsverkehrs zeigen. [Myths of e-invoices. How scientific findings show the way of implementing electronic invoicing]. Wissenschaft trifft Praxis 9(2): 70–80	Journal Paper	German
Binkow P	2015	The Impact of Self-Service Applications on Corporate Accounting and Its Customers. J. Corp. Acct. Fin 26(6): 81–85. https://doi.org/10.1002/jcaf.22084	Journal Paper	English
Bowles M, Ghosh S, Thomas L	2020	Future-Proofing Accounting Professionals: Ensuring Graduate Employability and Future Readiness. Journal of Teaching and Learning for Graduate Employability 11(1): 1–21	Journal Paper	English
Chen H, Yan Huang S, Chiu A, Pa, F	2012	The ERP system impact on the role of accountants. Industr Mngmnt & Data Systems 112(1): 83–101. https://doi.org/10.1108/02635571211193653	Journal Paper	English
Cong Y, Du H, Vasarhelyi MA	2018	Technological Disruption in Accounting and Auditing. Journal of Emerging Technologies in Accounting 15(2): 1–10. https://doi.org/10.2308/jeta-10640	Journal Paper	English
Crookes L, Conway E	2018	Technology Challenges in Accounting and Finance. In: Conway E, Byrne, D (eds) Contemporary Issues in Accounting. Palgrave Macmillan, Cham, pp 61–84	Chapter	English
Drerup B, Suprano F, Wömpener A	2018	Controller 4.0. Anforderungsprofil des Controllers im digitalen Zeitalter. [Controller 4.0. Profile of requirements for controllers in the digital age]. In: Horváth P, Reichmann T, Baumöl U, Hoffjan A, Möller K, Pedell B (eds) Umbrüche durch VUCA-Umfeld und Digitalisierung. Controlling Sonderheft. München, Vahlen, pp 13–18	Whitepaper	German
Egle U, Keimer I	2017	Digitaler Wandel im Controlling. Schriften aus dem Institut für Finanzdienstleistungen. IFZ, Zug. [Digital change in controlling. Papers from the Institute for financial services Zug. IFZ, Zug.]	Whitepaper	German
Egle U, Frisan A, Steiner M	2020	Digitaler Wandel im Controlling bei der Alpiq Gruppe. [Digital change in controlling of the Alpiq Group]. In: Keimer I, Egle U (eds) Die Digitalisierung der Controlling-Funktion: Anwendungsbeispiele aus Theorie und Praxis. Springer Gabler, Wiesbaden, pp 189–98	Chapter	German
Faustino Bauer M, Schulte M, Schwab JB	2019	Was Blockchain für das Accounting bedeutet. [What blockchain means for accounting]. Control Manag Rev 63(5):40–45. https://doi.org/10.1007/s12176-019-0025-6	Whitepaper	German
Fordham DR, Hamilton CW	2019	Accounting Information Technology in Small Businesses: An Inquiry. Journal of Information Systems 33(2):63–75. https://doi.org/10.2308/isys-51982	Journal Paper	English
Fuller SH, Markelevich A	2020	Should accountants care about blockchain? J. Corp. Acct. Fin 31(2):34–46. https://doi.org/10.1002/jcaf.22424	Journal Paper	English
Gadatsch A	2020	Grundkurs Geschäftsprozess-Management. [Business process management 101]. Springer, Wiesbaden	Chapter	German
Grönke K, Heimel J	2015	Big Data im CFO-Bereich—Kompetenzanforderungen an den Controller. [Big Data in the scope of the CFO. Competence requirements for controllers]. CON 27(4–5):242–48. https://doi.org/10.15358/0935-0381-2015-4-5-242	Whitepaper	German
Groß, C, Pfennig R	2019	Branchenübergreifende Anwendungen. [Applications accross industry sectors]. Gabler, Wiesbaden	Book	German
Hecht N, Scherrer P	2020	Nutzen und Stolpersteine bei der Einführung einer Business Intelligence-Lösung für KMU am Beispiel der Firma SIGA. [Benefits and stumbling blocks for the introduction of business intelligence solutions in small and medium-sized businesses by the example of the SIGA company]. In: Keimer I, Egle U (eds) Die Digitalisierung der Controlling-Funktion: Anwendungsbeispiele aus Theorie und Praxis. Springer Gabler, Wiesbaden, pp 83–102	Chapter	German
Heupel T, Lange W	2019	Wird der Controller zum Data Scientist? Herausforderungen und Chancen in Zeiten von Big Data, Predictive Analytics und Echtzeitverfügbarkeit. [Does the controller become a data scientist? Challenges and opportunities in times of big data, predictive analytics and real-time availability]. In: Hermeier B, Heupel T, Fichtner-Rosada, S (eds) Arbeitswelten der Zukunft: Wie die Digitalisierung unsere Arbeitsplätze und Arbeitsweisen verändert. FOM-Edition. Springer Gabler, Wiesbaden, pp 201–221. https://doi.org/10.1007/978-3-658-23397-6_12	Chapter	German
Hmyzo E Muzzu A	2020	Technologie im Rechnungswesen – Wenn die Maschine besser und schneller bucht. [Technology in accounting. When the machine transfers better and faster]. In: Berding F, Jahncke H, Slopinski A (eds) Moderner Rechnungswesenunterricht 2020: Status Quo und Entwicklungen aus wissenschaftlicher und praktischer Perspektive. Springer, VS, Wiesbaden, pp 99–113	Chapter	German
Janvrin DJ Weidenmier Watson M	2017	“Big Data”: A new twist to accounting. Journal of Accounting Education 38:3–8. https://doi.org/10.1016/j.jaccedu.2016.12.009	Journal Paper	English
Jonen A	2020	Aktuelle Trends und zukünftige Potenziale der Digitalisierung im Beschaffungscontrolling. [Current trends and future potentials of digitalisation in procurement controlling]. In: Keimer I, Egle U (eds) Die Digitalisierung Der Controlling-Funktion: Anwendungsbeispiele Aus Theorie Und Praxis. Springer Gabler, Wiesbaden, pp 349–72. https://doi.org/10.1007/978-3-658-29196-9_20	Chapter	German
Jordanski G	2020	Kaufmännische Steuerung und Kontrolle im 4.0 Arbeitsumfeld – Anforderungen an duale Ausbildungsberufe. [Commercial management and controlling in a 4.0 workfield]. In: Berding F, Jahncke H, Slopinski A (eds) Moderner Rechnungswesenunterricht 2020: Status Quo und Entwicklungen aus wissenschaftlicher Und praktischer Perspektive. Springer VS, Wiesbaden, pp 59–82	Chapter	German
Keimer I, Egle U	2020	Digital Controlling – Grundlagen für den erfolgreichen digitalen Wandel im Controlling. [Digital controlling. Fundamentals of a successfull digital transformation in controlling]. Springer Gabler, Wiesbaden	Book	German
Kink N	2007	Umfassende Controllingunterstützung. [Extensive controlling support]. Z Control Manag 51(5): 303–5. https://doi.org/10.1007/s12176-007-0083-z	Whitepaper	German
Kirchberg A	2017	Harmonisierung des externen und des internen Rechnungswesensaus aufbau- und ablauforganisatorischer Sicht. [Harmonisation of external and internal accounting from a workflow management and organisational structure point of view]. In: Klein A, Gräf A (eds) Reporting und Business Intelligence.. 3^rd^ edn Haufe Gruppe, Freiburg, München, Stuttgart, pp 85–102	Chapter	German
Klein J, Küst C	2020	Wie die Digitalisierung im Rechnungswesen die Aufgaben und Anforderungen an die Mitarbeiter/-innen verändert [How digitialisation in accounting changes the tasks and requirements of employees]. In: Berding F, Jahncke H, Slopinski A (eds) Moderner Rechnungswesenunterricht 2020: Status Quo und Entwicklungen aus wissenschaftlicher und praktischer Perspektive. Springer VS, Wiesbaden, pp 83–97	Chapter	German
Koch B	2017	Die E-Rechnung steht im Zeichen großer Marktveränderungen. Studie E-Rechnung. [E-invoices will bring about changes in the market]. Available via https://www.billentis.com/Marktstudie2017_elektronische_Rechnung.pdf. Accessed 21 Mar 2023	Whitepaper	German
Kokina J, Gilleran R, Blanchette S, Stoddard D	2021	Accountant as Digital Innovator: Roles and Competencies in the Age of Automation. Accounting Horizons 35(1):153–84. https://doi.org/10.2308/HORIZONS-19-145	Journal Paper	English
Kreher M	2021	Digitalisierung im Rechnungswesen 2021. [Digitalisation in accounting 2021]. KPMG. Available via https://home.kpmg/de/de/home/themen/2021/09/digitalisierung-im-rechnungswesen-2021.html. Accessed 05 Oct 2022	Whitepaper	German
Langmann C, Turi D	2020	Robotic Process Automation (RPA) – Digitalisierung und Automatisierung von Prozessen. Voraussetzungen, Funktionsweise und Implementierung am Beispiel des Controllings und Rechnungswesen. [Robotic process automation (RPA) – Digitalisation and automation of processes. Prerequisites, functions and implementation by the example of controlling and accounting]. Springer Gabler, Wiesbaden	Book	German
Le Guyader LP	2020	Artificial intelligence in accounting: GAAP's “FAS133”. J. Corp. Acct. Fin 31(3):185–89. https://doi.org/10.1002/jcaf.22407	Journal Paper	English
Losbichler H, Gänßlen S	2018	Performance Measurement in Zeiten von Big Data. Auswirkungen auf Kennzahlen und deren Reporting. [Performance measurement in times of big data. Implications on operating figures and their reporting]. In: Horváth P, Reichmann T, Baumöl U, Hoffjan A, Möller K, Pedell B (eds) Umbrüche durch VUCA-Umfeld und Digitalisierung. Controlling Sonderheft. München, Vahlen, pp 31–37	Whitepaper	German
Menges T	2012	Implementierung eines automatisierten Rechnungseingangs [Implementation of automated invoice reception]. In: Deickert F (ed) Erfolgsfaktor strategisches Management, Controlling und Personal: Zukunft des Gesundheitswesens. Mannheimer Schriften zur Gesundheitswirtschaft 3. Centaurus, Freiburg (Breisgau), pp 115–125. https://doi.org/10.1007/978-3-86226-855-9_8	Chapter	German
Mertens P	2015	Industrie 4.0 – Herausforderungen auch an Rechnungswesen und Controlling im Überblick. [Industry 4.0. Overview of challenges for accounting and controlling]. CON 27 (8–9):452–54. https://doi.org/10.15358/0935-0381-2015-8-9-452	Whitepaper	German
Müller A, Graumann M, Weiß HJ	2020	Innovationen für eine digitale Wirtschaft. Wie Unternehmen den Wandeln meistern. [Innovations for a digital economy. How companies succeed in the transformation]. Springer Gabler, Wiesbaden	Journal Paper	German
Müller R, Reichmann T	2010	Rechnungswesen und Controlling: Integrierte Softwarelösungen für den Mittelstand. [Accounting and controlling: integrated software solutions for small and medium-sized businesses]. CON 22(3):204–206. https://doi.org/10.15358/0935-0381-2010-3-204	Whitepaper	German
Nagel L	2018	Die Einführung der “E-Rechnung” bei den Kommunen gemäß EU-Richtlinie. Bachelorarbeit. [The introduction of e-invoices in local communities according to EU law. Bachelor thesis]. Hochschule für öffentliche Verwaltung und Rechtspflege. Available via https://opus.bsz-bw.de/hsf/files/119/bachelorarbeit_lisa_nagel.pdf. Accessed 21 Mar 2023	University Publication	German
Najderek A	2020	Auswirkungen der Digitalisierung im Rechnungswesen – ein Überblick. [Impications of digitalisation for accounting—an overview]. In: Müller A, Graumann M, Weiß H-J (eds) Innovationen für eine digitale Wirtschaft: Wie Unternehmen den Wandel meistern. Springer Gabler, Wiesbaden, pp 127–45	Chapter	German
Nwokike FO, Eya GM	2015	A Comparative Study of the Perceptions of Accounting Educators and Accountants on Skills Required of Accounting Education Graduates in Automated Offices. World Journal of Education 5(5):64–70. https://doi.org/10.5430/wje.v5n5p64	Journal Paper	English
Pagel P	2019	Bei der Übermittlung und Verarbeitung elektronischer Rechnungen müssen die geltenden Anforderungen an Datenschutz und Datensicherheit erfüllt sein. [The transmission and handling of electronic invoices needs to follow current regulations of data protection and safety]. Wirtsch Inform Manag 11(5):342–46. https://doi.org/10.1365/s35764-019-00201-w	Journal Paper	German
Pampel JR	2018	Digitale Horizonterweiterung. Begleitung der Innovation von Geschäftsmodellen durch das Controlling. [Broadening one's horizon. Accompanying innovative business models through controlling]. In: Horváth P, Reichmann T, Baumöl U, Hoffjan A, Möller K, Pedell B (eds) Umbrüche durch VUCA-Umfeld und Digitalisierung. Controlling Sonderheft. Vahlen, München, pp 21–29	Whitepaper	German
Pan G, Seow P-S	2016	Preparing Accounting Graduates for Digital Revolution: A Critical Review of Information Technology Competencies and Skills Development. Singapore Management University, Singapore	University Publication	English
Peters P	2006	Standardisierung im Rechnungswesen – Leistungssprung durch Shared Services. [Standardizing accounting. A performance leap through shared services]. Z. Control. Manag. 50(S8):94–100. https://doi.org/10.1365/s12176-006-0606-z	Whitepaper	German
Raschig S, Schulze M	2020	Weiterentwicklung des Finanz-Forecasts im Rahmen der digitalen Transformation am Beispiel der SAP SE. [Development of the financial forecast as part of digital transformation by the example of SAP SE]. In Keimer I, Egle U (eds) Die Digitalisierung der Controlling-Funktion: Anwendungsbeispiele aus Theorie und Praxis. Springer Gabler, Wiesbaden, pp 25–42	Chapter	German
Sandner P, Lange A, Schulden P	2020	The Role of the CFO of an Industrial Company: An Analysis of the Impact of Blockchain Technology. Future Internet 12(128):1–16. https://doi.org/10.3390/fi12080128	Journal Paper	English
Satzger G, Holtmann C, Peter S	2018	Advanced Analytics im Controlling. Potenzial und Anwendung für Umsatz- und Kostenprognosen. [Advanced analytics in controlling. Potencials and applications for sales and cost projections]. In: Horváth P, Reichmann T, Baumöl U, Hoffjan A, Möller K, Pedell B (eds) Umbrüche durch VUCA-Umfeld und Digitalisierung. Controlling Sonderheft. Vahlen, München, pp 47–53	Whitepaper	German
Schindera F, Jux M, Glustin O	2018	Banken-Controlling im digitalen Zeitalter. [Controlling in financial institutes in the digital age]. In: Horváth P, Reichmann T, Baumöl U, Hoffjan A, Möller K, Pedell B (eds) Umbrüche durch VUCA-Umfeld und Digitalisierung. Controlling Sonderheft. Vahlen, München, pp 61–67	Whitepaper	German
Schömburg H, Breitner MH	2010	Elektronische Rechnungen zur Optimierung der Financial Supply Chain: Status Quo, empirische Ergebnisse und Akzeptanzprobleme. [Electronic invoices to optimize the financial supply chain: current state, empirical results and acceptance issues]. MKWI 2010 – E-Commerce und E-Business:1253–64. Available via http://webdoc.gwdg.de/univerlag/2010/mkwi/03_anwendungen/e-commerce_und_e-business/07_elektronische_rechnungen_zur_optimierung_der_financial_supply_chain.pdf. Accessed 21 Mar 2023	Journal Paper	German
Selb M	2020	Von der Erfolgssicherung zur Produktentwicklung – Datenanalyse bei Gebrüder Weiss im Fachbereich Corporate Logistics. [From securing long-term success to product development—Data analysis of Gebrüder Weiss in the area of corporate logistics]. In: Keimer I, Egle U (eds) Die Digitalisierung der Controlling-Funktion: Anwendungsbeispiele aus Theorie und Praxis. Springer Gabler, Wiesbaden, pp 43–64	Chapter	German
Shah H, Jiles L	2020	Management accountants and other professionals are leveraging data analytics to react effectively to the COVID-19 pandemic. A data-driven approach to the pandemic. Strategic Finance, 26–33	Whitepaper	English
Sinzig W	2015	In-memory Technik für Rechnungswesen und Controlling. [In-memory technique for accounting and controlling]. CON 27 (4–5):236–241. https://doi.org/10.15358/0935-0381-2015-4-5-236	Whitepaper	German
Sledgianowski D, Gomaa M, Tan C	2017	Toward integration of Big Data, technology and information systems competencies into the accounting curriculum. Journal of Accounting Education 38:81–93. https://doi.org/10.1016/J.JACCEDU.2016.12.008	Journal Paper	English
Spraakman G, O'Grady W, Askarany D, Akroyd C	2015	Employers' Perceptions of Information Technology Competency Requirements for Management Accounting Graduates. Accounting Education 24(5):403–422	Journal Paper	English
Suden PT	2010	Einführung elektronischer Rechnungen. [Integration of electronic invoices]. Gabler, Wiesbaden	Book	German
Tanner C	2016	E-Invoicing – ein erster Schritt zur digital vernetzten Wirtschaft. [E-invoicing. A first step to a digitally cross-linked economy]. Die Fachzeitschrift für erfolgreiche Unternehmer und Top-Manager (KMU Magazin) 19(4):44–48. https://doi.org/10.26041/fhnw-649	Whitepaper	German
Tanner C, Wölfle R	2005	Elektronische Rechnungsstellung zwischen Unternehmen. Konzentriertes Wissen aus der swissDIGIN-Initiativezur Förderung des elektronischen Rechnungsaustausch. [Electronic invoicing between companies. Concentrated knowledge from the swissDIGIN initiative for the promotion of electronic invoicing]. Available via https://unece.org/fileadmin/DAM/cefact/forum_grps/tbg/tbg15/project_docs/swissDIGIN_Leitfaden.pdf. Accessed 21 Mar 2023	University Publication	German
Trachsel V, Bitterli C	2020	Controller-Profile in der Schweiz. Bedeutung der Digitalisierung. [Profiles of controlling employees in Switzerland. The meaning of digitalisation]. In: Keimer I, Egle U (eds) Die Digitalisierung der Controlling-Funktion: Anwendungsbeispiele aus Theorie und Praxis. Springer Gabler, Wiesbaden, pp 199–210	Chapter	German
Weiber R, Gassler H, Meyer J	2002	Qualifizierungsanforderungen im E-Business [Qualification requirements in e-business]. In: Weiber R (ed) Handbuch Electronic Business: Informationstechnologien – Electronic Commerce – Geschäftsprozesse. 2^nd^ edn. Gabler, Wiesbaden, pp 261–77	Chapter	German
Werner M, Gehrke N	2011	Potentiale und Grenzen automatisierter Prozessprüfungen durch Prozessrekonstruktionen. [Potentials and boundaries of automated process verification through process reconstruction]. In: Plate G (ed) Forschung für die Wirtschaft. pp 99–120	Chapter	German
Wettstein K, Caderas R	2020	Die Digitale Transformation des Reportings beim Schweizer Radio und Fernsehen (SRF). [The digital transformation of reporting at the Swiss Radio and Television organisation (SRF)]. In: Keimer I, Egle, U (eds) Die Digitalisierung der Controlling-Funktion: Anwendungsbeispiele aus Theorie und Praxis. Springer Gabler, Wiesbaden, pp 65–82	Chapter	German
Wilczek T	2014	Elektronische Rechnungseingangsbearbeitung. Eine Make-or-Buy-Studie. [Electronic reception of invoices and their processing. A make-or-buy study]. Disserta, Hamburg	University Publication	German

## Abbreviations

AI: Artificial intelligence
ERP: Enterprise resource planning
OCR: Optical character recognition
RPA: Robotic process automation
RQ: Research question
